# Attention and Memory Biases as Stable Abnormalities Among Currently Depressed and Currently Remitted Individuals with Unipolar Depression

**DOI:** 10.3389/fpsyt.2012.00099

**Published:** 2012-11-23

**Authors:** Rashmi Gupta, Bhoomika R. Kar

**Affiliations:** ^1^Centre of Behavioral and Cognitive Sciences, University of AllahabadAllahabad, India; ^2^Institute of Cognitive Neuroscience, University College LondonLondon, UK

**Keywords:** currently depressed, currently remitted individuals with unipolar depression, attention bias, memory bias, stable markers

## Abstract

**Background:** In the present study, we explored the possibility of the stability of attention bias and memory bias in currently remitted individuals with unipolar depression compared to currently depressed individuals with unipolar depression and never-depressed individuals. **Methods:** The Emotional Stroop and autobiographical memory task (AMT) were administered on 10 participants, who were currently depressed, currently remitted with unipolar depression, or never-depressed. In the emotional Stroop task (EST), the respondent’s task was to indicate the color of the ink of the positive, negative, and neutral words by selecting one of a series of colored blocks. In the AMT, participants were presented with positive, negative, and neutral cue words. For each word, they were asked to report specific events from their life. **Results:** Both the attention bias and memory bias exist in both the clinical groups. In EST, both currently depressed and currently remitted groups were slower to respond to negative words compared to neutral words. Unlike EST, in AMT both currently depressed and currently remitted groups were slower to respond to positive words compared to neutral words. Interestingly, the capacity to generate specific events for negative events was higher in both currently depressed and currently remitted groups. They were over-general in their memories of positive events. Importantly, the never-depressed group was specific in their memories of both positive and negative events of their life. **Conclusion:** Our findings provide evidence for the stable existence of attention and memory bias in currently remitted individuals. This study has implications for the cognitive behavior therapy for depression to include modules to resolve the attention and memory bias toward negative thought and content, and to build strategies to overcome such biases.

## Introduction

Cognitive processes like selective attention and memory influence the affective states and vice versa. Greater allocation of these cognitive processes to a particular category of stimuli is referred to as cognitive bias. The question of cognitive biases such as attention bias and memory bias has long held interest in the study of cognitive mechanisms underlying depression. These biases are critical aspects of several cognitive theories of depression (Beck, 1967; Abramson et al., 1978, 2002; Bohon et al., 2008; Hankin, 2008; Meites et al., 2008). These theories suggest that the abnormal attentional processes in depression affect the unintended, involuntary encoding of self-referent material. This effect on encoding is the product of activated schemas involving loss, failure, and deprivation, and could lead to the development of mood disorders. More specifically, Beck contends that negative schemas that characterize individuals who are at elevated risk for depression are activated in the face of negative life events, leading to clinically significant depression.

The cognitive theory also postulates cognitive biases as predisposing or susceptibility factors to depression. Cognitive biases persist beyond the depressive state, which in turn may cause the relapse of depressive state. Consistent with Beck’s (1967) theory, some investigators have suggested that negatively biased cognitive processing in attention and memory may represent a stable vulnerability factor for unipolar depression, which may cause high rate of recurrence of depressive episodes (Gotlib and Cane, 1987; Joormann and Gotlib, 2007; Gupta and Kar, 2008). Hedlund and Rude (1995) found that formerly depressed individuals responded in a significantly more negative direction than never-depressed individuals on two information-processing tasks, scrambled sentences and incidental recall tasks, but not on a Stroop color-naming exercise. The researchers concluded that negative cognitive processing is not only concomitant to depression but that it can be demonstrated in depression-prone individuals.

Gilboa and Gotlib (1997) randomly assigned previously dysphoric and never-dysphoric participants to a positive and negative autobiographical mood induction condition. Although the previously dysphoric group showed more persistent dysphoria to the negative mood induction than the never-dysphoric group, the two groups did not differ significantly in their performance on an emotional Stroop color-naming task following negative mood induction. In another experiment, Gilboa and Gotlib (1997) again found that the previously dysphoric participants had more persistent dysphoria in response to the negative mood induction, but again failed to differ from the never-depressed in their Stroop color-naming performance. However, the previously dysphoric did recall more negative words than the never-dysphoric group, but these differences were independent of current mood state. The authors concluded that negatively biased recall and not attention or judgment might be a vulnerability marker for depression or dysphoria. Using a dot-probe task, Joormann and Gotlib (2007) examined attentional bias in the processing of emotional faces in currently and formerly depressed individuals and healthy controls. They found that both currently and formerly depressed individuals selectively attended to the sad faces, whereas the control group selectively avoided the sad faces and oriented toward the happy faces (positive bias). These results indicate that attentional bias for the processing of sad faces are evident even after individuals have recovered from a depressive episode.

It is also suggested that depressed individuals not only show memory bias in terms of the speed with which they can remember positive and negative events from their past, but they also find it difficult to be specific in their recall of events. This tendency has been found to be more evident with positive than negative events (Williams and Scott, 1988). Using implicit measures such as the Implicit Association Test, Meites et al. (2008) found the presence of a reduced tendency to associate self with happiness in the remitted depressive with unipolar depression group.

Most of the studies have provided evidence to support for the existence of cognitive biases in the currently remitted group. However, there are some studies that have failed to find persistence of negative processing bias or increased accessibility to negative cognitive constructs with recovery from depression (see Williams et al., 1997, for a review). For example, Segal and Gemar (1997) found a significant reduction in negative interference scores for self-referent adjectives on the primed emotional Stroop color-naming task in depressed patients who improved following cognitive therapy for depression. McCabe and Gotlib (1993) found that individuals who had recovered form their depression no longer exhibited a selective attentional effect from negative content adjectives in a focused-attention dichotic listening task. Gotlib and Cane (1987) also found that recovered depressed patients no longer showed color-naming interference to depressed-content words in an emotional Stroop task (EST).

Studies employing self-referent encoding tasks have again failed to find persistence of a bias to rate negative trait adjectives as more self-descriptive, or to show a selective recall bias for negative self-referent trait adjectives in remitted individuals with unipolar depression (Dobson and Shaw, 1987; Bradley and Mathews, 1988). However, Teasdale and Dent (1987) did find that in a neutral mood state, recovered depressed women endorsed more negative trait adjectives and recalled fewer positive words than never-depressed women. Hammen et al. (1986) also found that dysphoric students who showed an enhanced recall for negative self-reference words at Time 1 did not show this effect at Time 2 if they were no longer depressed.

The existing literature suggests that studies in the area of cognitive biases in remitted individuals with unipolar depression are very few and not very conclusive. It is important to note that, so far, attention and memory biases have been examined separately in different studies. No studies have investigated the co-occurrence of attention and memory bias in both currently depressed and currently remitted groups with unipolar depression. Furthermore, previous studies used different tasks or different methodology to comment on stability of cognitive biases in currently remitted individuals with unipolar depression. In addition, studies differ in their criteria for selecting remitted individuals. Use of different methodologies, criteria, and the fact that the two forms of bias have been examined in different groups of the population might lead to inconsistent patterns of results. Thus, it is not clear whether both biases co-exist in the same currently depressed individuals with unipolar depression, nor whether they are evident even after individuals have recovered from a depressive episode.

The present study was designed to address this issue by examining the attention and memory bias concurrently on currently depressed and currently remitted individuals with unipolar depression as compared to never-depressed individuals. Such a design will remove confounds such as difference in experimental procedures and participant demographics. In addition, it gives an opportunity to look at the possibility of the stability of attention and memory bias in currently remitted individuals with unipolar depression. An EST for attention bias and an autobiographical memory task (AMT) for memory bias were used with three groups of participants. Interestingly, it should be noted that as yet only one study has looked at the processing of autobiographical memory style in currently remitted individuals with unipolar depression (Mansell and Lam, 2004). Hence, our study throws some light on the processing style of autobiographical memory in these individuals.

## Materials and Methods

### Participants

Three groups of participants were employed in the study: currently depressed with unipolar depression (*N* = 10, females = 5), currently remitted individuals with unipolar depression (*N* = 10, females = 5), and never-depressed individuals (*N* = 10, females = 5). All participants were between 21 and 53 years of age and their primary language was Hindi. Both clinical groups were recruited through the Department of Psychiatry, Swaroop Rani Medical College and Hospital in Allahabad city. All participants were invited for a Structured Clinical Interview for DSM-IV (SCID; First et al., 2002). Structured interview and clinical history was taken by a licensed mental health counselor.

The selection criteria for currently depressed group were (a) primary diagnosis of major depression according to Diagnostic and Statistical Manual of Mental Disorders (DSM-IV; American Psychiatric Association, 1994) criteria (the diagnosis was determined in a clinical interview at the end of the administration of the tasks) and (b) a score of >17 on the Hamilton Depression Rating Scale (HDRS; Hamilton, 1960). Participants with a history of unipolar depression but no longer meeting DSM-IV criteria and with a score of ≤7 on HDRS were included as currently remitted individuals with unipolar depression (Bell and Rothschild, 2004). Participants with bipolar disorder, psychiatric/neurological disorder, clinical evidence of mental retardation, and having motor, speech deficits were excluded from the study. Both the clinical groups were only on antidepressant medication, but not on other therapies such as cognitive behavioral therapy or electroconvulsive therapy. They were asked to interrupt medication the day before and on the day that testing occurred. Never-depressed participants who scored ≤3 on General Health Questionnaire (GHQ) were included (Goldberg, 1978). GHQ was used to detect psychiatric disorders among never-depressed participants. The participants were also matched on gender, age, and expressive speech. Brief tests were administered to rule out vision, hearing, attention, and expressive speech deficits for all the three groups. Expressive speech was assessed using tests of repetitive speech, nominative speech and narrative speech in question answer form. Purpose of this test was rule out speech related problems in the participants. None of the participants showed speech related difficulties. Further characteristics related to participants are presented in Table 1.

**Table 1:** **Demographic characteristics of the currently depressed group with unipolar depression, (CD), currently remitted group with unipolar depression (RD), and never-depressed group (ND)**.

Characteristics	CD (*n* = 10)			RD (*n* = 10)			ND (*n* = 10)		
	Range	*M*	SD	Range	*M*	SD	Range	*M*	SD
Age (years)	40.0	8.37	21–50	43.0	4.49	38–53	43.60	5.21	32–50
Education (years)	16.50	4.30	–	15.70	3.33	–	18.30	2.11	–
Age at onset (years)	37.5	8.5	21–49	37.9	5.9	28–49			–
Duration of remission	–	–	–	4 months	0.18	3–9 months	–	–	–
HDRS^i^	22.8	2.9	18–24	6.00	1.4	4–9	4.3	0.48	4–5
GHQ^R^	–	–	–	–	–	–	1.6	0.69	1–3

*^i^Hamilton depression rating scale; ^R^General health questionnaire*.

#### Measures

##### Screening tools

*Hamilton depression rating scale*. The HDRS (Hamilton, 1960) was used to select participants for the study as well as to assess the severity of depression in currently depressed and currently remitted individuals with unipolar depression. The HDRS consists of 21 items, each of which is rated 0–4 or 0–2, with a maximum total range of 0–76. The ratings were derived from a structured clinical interview with the participants. Answers to questions about feelings of guilt, suicide, sleep habits, and other symptoms of depression were elicited. The total scores inform about the level of severity of depression, where a score in the range of 0–6 falls in the level indicating no depression, 7–17: mild depression, 18–24: moderate level of depression, and 25-above indicates severe depression. The HDRS has shown acceptable levels of both validity (Carroll et al., 1973) and inter-rater reliability (Bech et al., 1975).

*General health questionnaire*. The 12-item scale of GHQ (Goldberg, 1978) was used to detect psychiatric disorders among never-depressed participants. GHQ is a self-administered screening test used to rule out psychiatric disturbances among respondents in community settings and neuropsychiatric clinical settings. It is found to be highly reliable. The cut-off score of ≤3 on the GHQ indicates absence of any behavioral disturbances.

##### Tasks for cognitive biases

**Figure 1:**
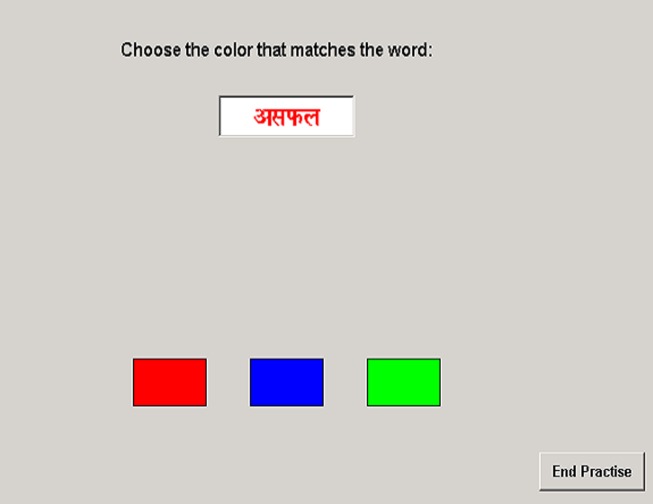
**An example of the emotional stroop task**.

*Attention bias emotional stroop task*. The EST was developed to examine attention bias. The EST consisted of three rectangular blocks (2.5 cm × 1.5 cm) of red, blue, and green respectively which appeared simultaneously on the screen (14″ monitor, 800 × 600 pixels resolution, 32-bit color). At the same time a white rectangular block (5.0 cm × 1.5 cm) which contained a word (1.00 cm high) appeared above the three colored blocks (see Figure 1). The words were randomly sampled from a set of four positive, four negative, and four neutral words in three different colors (red, green, and blue), with 180 total presentations. The stimulus words were selected using the following procedure. A list of words was taken from the Brittlebank et al. (1993). Selection of the words was determined by the length of the word and their frequency. Words were translated into Hindi to avoid any interference effect of secondary language on performance. To validate translation, two translators performed the English-to-Hindi translation and two others performed the Hindi-to-English back translation. The translators were not aware of the purpose of the study. All the words were then presented to 10 raters who were unaware of the purpose of the task. They were asked to rate each of the positive, negative, and neutral words for the positive, negative, or neutral content of the word respectively on a three-point rating scale. Percentage of agreement across judges was tabulated, and only those words that achieved at least ≥60% agreement were selected for the task.

The respondent’s task was to match the color of the ink of the word with the color of the block: they were required to click on the colored block which matched the color of the ink of the word as soon as possible while ignoring the verbal content of the word. Exposure time of the word was dependent upon time taken to respond (reaction time). There were 180 such trials, preceded by 30 practice trials.

*Memory bias: autobiographical memory task*. The AMT (Williams and Broadbent, 1986) was conducted to investigate memory bias. The AMT is a sensitive test to assess memory bias in depression (Williams and Scott, 1988). The AMT comprised of 15 cue words: five positively toned words, five negatively toned words, and five emotionally neutral words. The selection procedure of 15 cue words was same as described in the EST. Each word was read one by one to the participants in a random order. Participants were asked to narrate a life event related to that cue word. The responses to each cue word in terms of narrations of episodic events were recorded. If the participants did not respond specifically in terms of narrating an event in response to the cue word within 60 s, that fact was recorded and the investigator read out the next word. Time taken to respond to the word and specificity of the autobiographical recall were the two measures of performance on this test. The reaction time to respond to each word on the list was recorded using a stopwatch. The stopwatch operator was unaware with the purpose of the study. If the participants gave a general comment then they were prompted to be very specific in recalling and narrating the event/episode. The experimenter asked them to describe the memory, trying to recreate the scene, or impression in as much detail as possible, describing what they saw, heard, or felt. Each memory was recorded on audiotape. An event was considered specific if the participant was later able to give a date, day of the week, or time of the day when the event/episode occurred, or general, if they described a series of events over a period in their life or a type of event (see Williams and Broadbent, 1986; Williams and Scott, 1988; Mansell and Lam, 2004 for detailed discussion). A sample of 15 of the memories (five positive, five negative, and five neutral) were coded independently by the experimenter and the second author (who was blind to the experimental group). They met to discuss discrepant codings and these were recorded by discussion of the appropriate criteria. The experimenter then coded the remaining memories (as carried out by Williams and Broadbent, 1986) and the second author coded a sample of 15 of these to check for inter-rater reliability (see also Mansell and Lam, 2004).

#### Procedure

Following informed consent, participants were screened using appropriate measures, followed by administration of the EST and AMT with adequate rest pauses. The order of the tasks was counterbalanced. Finally, all participants underwent the structured clinical interview on the HDRS and were debriefed. The present study has been approved from the ethical committee of the Centre of Behavioral and Cognitive Sciences, University of Allahabad, India.

#### Statistical analysis

For each score, data were submitted to 3(Group: currently depressed, currently remitted, never-depressed) × 3(word valence: positive, negative, neutral) mixed factor design, where Group was a between group factor and word valence was a within group factor. In general, clinical groups showed more variability in their response compared to never-depressed group.

## Results

### Sample characteristics

Demographic characteristics are presented in Table 1. Age and education were not significantly different across groups. The currently depressed group scored significantly higher than the other two groups on the HDRS and they were significantly different from the currently remitted group with unipolar depression, *F*(1, 18) = 265.7, *p* < 0.001, and from the never-depressed group, *F*(1, 18) = 386.48, *p* < 0.001, on severity of depression. The currently remitted group with unipolar depression were not significantly different from the never-depressed group on the HDRS, suggesting that the clinical condition of these participants did not qualify for a diagnosis of major depression at the time of assessment and that they were symptom free (see Table 1).

### Emotional stroop task

The two-way ANOVA revealed a significant main effect of group, *F*(2, 27) = 6.872, *p* < 0.01, with larger latencies for the currently depressed (*M* = 2.08 s), *F*(1, 27) = 4.8, *p* < 0.001, and currently remitted groups (*M* = 1.90 s), *F*(1, 27) = 4.08, *p* < 0.01, as compared to never-depressed group (*M* = 0.77 s). There was a significant interaction effect between group × word valence, *F*(4, 54) = 4.54, *p* < 0.01.

**Figure 2:**
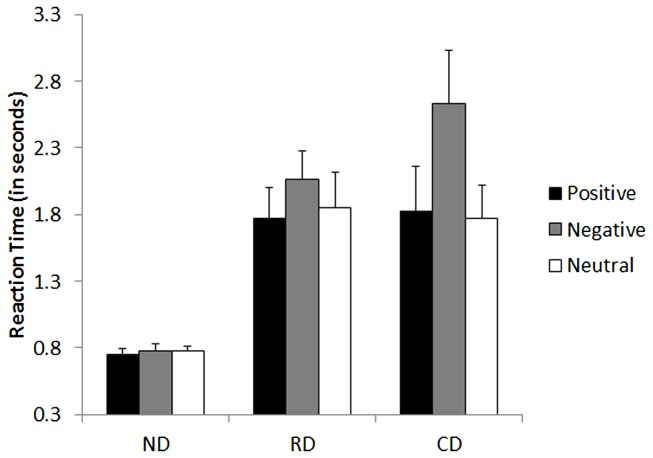
**Mean performance of currently depressed group with unipolar depression, currently remitted group with unipolar depression, and never-depressed group on the emotional stroop task of attention bias**. CD, currently depressed group with unipolar depression; RD, currently remitted group with unipolar depression; ND, never-depressed group.

*Post hoc* analysis indicated that both the currently depressed, *t*(1, 9) = 2.27, *p* < 0.05, and currently remitted groups, *t*(1, 9) = 3.39, *p* < 0.01, were significantly slower to respond for negative words compared to neutral words. There was no significant difference in reaction time to respond for negative and neutral words in the never-depressed group, *t*(1, 9) = 0.192, *p* = 0.85. There was no significant difference in reaction time to respond for positive words compared to neutral words in the currently depressed, currently remitted, or never-depressed group (*p* > 0.16, for all). These results indicated that both the clinical groups showed attention bias to respond for negative words (see Figure 2).

### Autobiographical memory task

#### Response latencies

The two-way ANOVA revealed a significant main effect of word valence, *F*(2, 27) = 11.26, *p* < 0.001, with significantly higher response latencies for positive words(*M* = 13.43 s) when compared to both negative (*M* = 10.67 s), *F*(1, 54) = 4.2, *p* < 0.01, and neutral (*M* = 9.63 s), *F*(1, 54) = 5.6, *p* < 0.001 cue words. There was a significant interaction effect between group and word valence, *F*(4, 54) = 12.82, *p* < 0.001.

**Figure 3:**
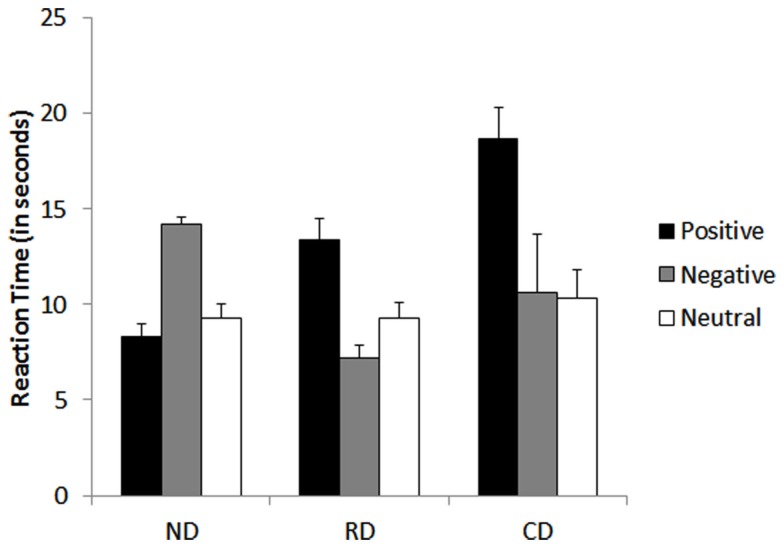
**Mean performance of currently depressed group with unipolar depression, currently remitted group with unipolar depression, never-depressed group on the autobiographical memory task of memory bias**. CD, currently depressed group with unipolar depression; RD, currently remitted group with unipolar depression; ND, never-depressed group.

*Post hoc* comparisons indicated that both the currently depressed, *t*(1, 9) = 4.91, *p* < 0.001, and currently remitted groups, *t*(1, 9) = 2.82, *p* < 0.05, were significantly slower to respond for positive cue words compared to neutral cue words. In contrast, the never-depressed group were slower to respond to negative cue words than neutral, *t*(1, 9) = 6.25, *p* < 0.001. There was no significant difference in reaction time to respond for negative cue words compared to neutral cue words in the currently depressed group, *t*(1, 9) = 0.120, *p* = 0.90. Interestingly, the currently remitted group responded faster for negative words compared to neutral words, *t*(1, 9) = 2.93, *p* < 0.01. There was no significant difference in reaction time between positive and neutral cue words in never-depressed group, *t*(1, 9) = 0.856, *p* = 0.41. These results indicated that negative bias for recalling events from memory was found in both the currently depresses and currently remitted groups, and interestingly both clinical groups appear to have lost the positive memory bias that characterized never-depressed group (see Figure 3).

#### Memory specificity

The two-way ANOVA revealed a significant main effect of group, *F*(2, 27) = 9.50, *p* < 0.001, with memories of both the currently depressed group (*M* = 53.3%), *F*(1, 27) = 5.3, *p *< 0.001, and the currently remitted group (*M* = 53.3%), *F*(1, 27) = 5.3, *p* < 0.001, being less specific than those of never-depressed group (*M* = 70%). The main effect of cue valence was also significant, *F*(2, 27) = 106.82, *p* < 0.001, with less specific responses to positive, *F*(1, 54) = 11.0, *p* < 0.001, and neutral cue words, *F*(1, 54) = 20.6, *p* < 0.001, compared to negative cue words. Memory specificity was highest for negative cue words (*M* = 83.3%) followed by positive cue words (*M* = 57.7%) with worst specificity for neutral cue words (*M* = 35.5%). Interestingly, there was a significant interaction between group × cue valance, *F*(4, 54) = 16.15, *p* < 0.001.

**Figure 4:**
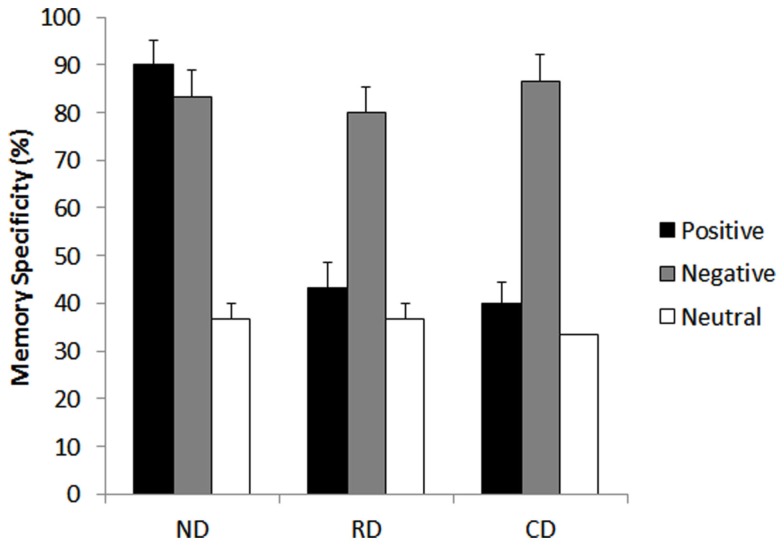
**Mean proportions of specific memories in percentage of currently depressed group with unipolar depression, currently remitted group with unipolar depression, and never-depressed group on the autobiographical memory task of memory bias**. CD, currently depressed group with unipolar depression; RD, currently remitted group with unipolar depression; ND, never-depressed group.

*Post hoc* analysis indicated that both the currently depressed group, *t*(1, 9) = 9.79, *p* < 0.001, and the currently remitted group, *t*(1, 9) = 8.5, *p* < 0.001, were more specific when recalling negative events than neutral. There was no significant difference in memory specificity between positive and neutral memory for either the currently depressed, *t*(1, 9) = 1.50, *p* = 0.168, or currently remitted groups, *t*(1, 9) = 1.5, *p* = 0.16. Interestingly, the never-depressed group was more specific in both positive, *t*(1, 9) = 9.79, *p* < 0.001, and negative memories, *t*(1, 9) = 8.57, *p* < 0.001, compared to neutral events (see Figure 4).

## Discussion

The present study was designed to investigate the possibility of stable and co-existing attention and memory biases in currently remitted individuals with unipolar depression. We hypothesized that if attention bias and memory biases both exist even after individuals have recovered from a depressive episode (currently remitted individuals with unipolar depression) then it gives an indication that both attention and memory biases are stable markers of depression.

Confirming our prediction, we found that attention and memory bias both exist in both clinical groups. We found that both currently depressed and currently remitted individuals with unipolar depression were slower to respond to negative words in the EST and slower as well as less specific in recalling positive episodic events of their life. These results indicate that both currently depressed and currently remitted individuals selectively attended to the negative information even though that was not related to the particular task at hand. In addition, both clinical groups appear to have lost the positive memory bias that characterized never-depressed individuals. These findings indicate that like currently depressed individuals, currently remitted individuals with unipolar depression might also not be able to allocate sufficient resources to cognize positive information. This result is consistent with a previous report that currently remitted individuals with unipolar depression were more specific in their recall for negative events compared to currently remitted individuals with bipolar depression (Mansell and Lam, 2004).

The clinical implications of limited capacity to generate specific positive memories could be that participants need help to access their positive memories, if such memories are available, or help to reinterpret negative memories in a more balanced manner (e.g., standard cognitive therapy techniques). Recent findings suggest that over-general memory may be reduced by cognitive interventions and maintained by ruminations (Watkins et al., 2000). Rumination is associated with the maintenance of over-general memory. Ruminative style is found in individuals at risk for depression (including people in remission) and in people who are currently depressed (Nolen-Hoeksema, 1991) and therefore may contribute to the high rates of over-general memory for positive cue words observed in the present study. These results indicate that even after the recovery of mood symptoms, currently remitted individuals with unipolar depression did not reach the normal levels of information-processing and appeared to be susceptible, in terms of the maintenance of cognitive biases. Previous studies also indicate that faulty cognitive processing is not only a concomitant of depression but it can be observed in currently remitted individuals with unipolar depression, even when they are not currently symptomatic of depression (Beck, 1984; Hankin, 2008; Meites et al., 2008). Existence of both attention and memory bias in both clinical groups strengthen the claim that low executive control is related to a reduction in autobiographical memory specificity (Dalgleish et al., 2007). Since the AMT has a time limit, over-generality would result from slowed cognitive processes or from a lack of inhibition, leading depressed patients to answer before having reached event-specific knowledge (Conwaym and Pleydell-Pearce, 2000).

It should be noted that from the current study, we cannot comment on whether abnormalities in both biases suggest underlying dysfunction in common brain systems or multiple brain systems. However, recently Disner et al. (2011) integrated the neurobiological and cognitive mechanism into a single model called the cognitive-neurobiological model of depression. In this model, they proposed that there are two key processes underlying cognitive biases in depression. First, neurobiological processes start the cognitive bias and reduced cognitive control, which allows bias to persist (hyper-activation of the bottom-up pathway). The second component takes the form of attenuation of the cognitive control (hypo-activation of the top-down pathway) that in healthy individuals prevents unrestrained activation in emotional regions of the brain. They suggested that negative cognitive biases in depression are facilitated by increased influence from subcortical emotion processing regions, combined with attenuated top-down cognitive control (see Disner et al., 2011, for a detailed discussion). This model seems to suggest that a “bottom-up” system could produce both of the cognitive biases which were observed in the present study, which might give an indication that both biases are different measures of a single underlying process. However, this possibility should be tested appropriately in future studies.

Present findings could have implications for treatment of depression. It appears that cognitive biases are more stable than was previously believed, and that these biases should be targeted through long-term specific interventions during, and even after, a depressive episode. Successful treatment of depression not only requires a successful resolution of depressed symptoms but, more importantly, change in the underlying, stable cognitive factors that increase susceptibility for repeated episodes. There are some reports which suggest that psychological treatment like cognitive behavioral therapy influence the later stage of information-processing, presumably via an effect on frontal control regions (Browning et al., 2010, for review). Therefore, management of depression should focus on reducing cognitive susceptibility to depression, with the inclusion of both the pharmacological and psychological treatment, rather than only targeting a reduction in the symptoms.

In conclusion, the present study is the first study which clearly indicates that both attention and memory bias simultaneously exist beyond the depressive episodes, which suggests that both cognitive biases are stable markers of depression. Further studies could be done to see the relationship between the two biases to understand the etiology of depression. There were some limitations of the present study. The sample size in the present study was small and restricts the wider generalization of the results; thus this should be considered as a preliminary study showing promising trends about stability of cognitive biases. In addition the GHQ was administered only on the never-depressed group, which was an experimenter error. However, clinical history shows that both the clinical groups did not show any general health problems. Certainly, a replication of the present results with a larger sample and with more stringent selection criteria taking medication into account is warranted.

Despite this limitation, our findings provide evidence of stability of attention and memory bias in currently remitted individuals with unipolar depression. This study has implications for future longitudinal studies, which could investigate whether the stability of both attention and memory biases in currently remitted individuals is predictive of relapse in unipolar depression. In addition, a longitudinal study will be helpful to explain the process as to how biases resolve from the state of severe depression through remission.

## Conflict of Interest Statement

The authors declare that the research was conducted in the absence of any commercial or financial relationships that could be construed as a potential conflict of interest.

## Abbreviations

AMT, autobiographical memory task; CD, currently depressed group with unipolar depression; DSM-IV, diagnostic and statistic manual of mental disorders; EST, emotional stroop task; GHQ, general health questionnaire; HDRS, Hamilton depression rating scale; ND, never-depressed group; RD, currently remitted group with unipolar depression.

## References

[B1] Abramson L. Y. Alloy L. B. Hankin B. L. Haeffel J. G. MacCoon D. G. Gibb B. E. (2002). “Cognitive vulnerability-stress models of depression in a self-regulatory and psychobiological context,” in Handbook of Depression, eds Gotlib I. H. Hammen C. L. (New York: Guilford), 268–294

[B2] Abramson L. Y. Seligman M. E. Teasdale J. D. (1978). Learned helplessness in humans: critique and reformulation. J. Abnorm. Psychol. 87, 49–74 10.1037/0021-843X.87.1.102 649856

[B3] American Psychiatric Association (1994). Diagnostic and Statistical Manual of Mental Disorders, 4th Edn Washington, DC: American Psychiatric Association

[B4] Bech P. Gram L. F. Dein E. Jacobsen O. Vitger J. Bolwig T. G. (1975). Quantitative rating of depressive states. Acta Psychiatr. Scand. 51, 161–170 10.1111/j.1600-0447.1975.tb00002.x 1136841

[B5] Beck A. T. (1967). Depression: Causes and Treatment. Philadelphia: University of Pennsylvania Press

[B6] Beck A. T. (1984). Cognition and therapy. Arch. Gen. Psychiatry 41, 1112–1114 10.1001/archpsyc.1983.01790220102019 6497575

[B7] Bell M. A. Rothschild A. J. (2004). Psychotic depression: state-of-the-art algorithm improves odds for remission. Curr. Psychiatr. 3, 54–63

[B8] Bohon C. Stice E. Burton E. Fudell M. (2008). A prospective test of cognitive vulnerability models of depression with adolescent girls. Behav. Ther. 39, 79–90 10.1016/j.beth.2007.05.003 18328873 PMC2773503

[B9] Bradley B. Mathews A. (1988). Memory bias in recovered clinical depressives. Cogn. Emot. 2, 235–246 10.1080/02699938808410926

[B10] Brittlebank A. D. Scott J. Williams J. M. G. Ferrier I. N. (1993). Autobiographical memory in depression: state of trait marker. Br. J. Psychiatry 162, 118–121 10.1192/bjp.162.1.118 8425125

[B11] Browning M. Holmes E. A. Harmer C. J. (2010). The modification of attentional bias to emotional information: a review of the techniques, mechanisms, and relevance to emotional disorders. Cogn. Affect. Behav. Neurosci. 10, 8–20 10.3758/CABN.10.1.8 20233952

[B12] Carroll B. J. Fielding J. M. Blashki T. G. (1973). Depression rating scales. A critical review. Arch. Gen. Psychiatry 28, 361–366 10.1001/archpsyc.1973.01750330049009 4688625

[B13] Conwaym M. A. Pleydell-Pearce C. W. (2000). The construction of autobiographical memories in the self-memory system. Psychol. Rev. 107, 261–288 10.1037/0033-295X.107.2.261 10789197

[B14] Dalgleish T. Williams J. M. G. Golden A.-M. J. Perkins N. Barrett L. F. Barnard P. J. (2007). Reduced specificity of autobiographical memory and depression: the role of executive control. J. Abnorm. Psychol. 136, 23–42 10.1037/0096-3445.136.1.23 PMC2225543 17324083

[B15] Disner S. G. Beevers C. G. Haigh E. A. P. Beck A. T. (2011). Neural mechanisms of the cognitive model of depression. Nat. Rev. Neurosci. 12, 467–477 10.1038/nrm3166 21731066

[B16] Dobson K. S. Shaw B. F. (1987). Specificity and stability of self-referent encoding in depression. J. Abnorm. Psychol. 96, 34–40 10.1037/0021-843X.96.1.34 3558947

[B17] First M. B. Spitzer R. L. Gibbon M. Williams J. B. W. (2002). Structured Clinical Interview for DSM-IV-TR Axis I Disorders, Research Version, Patient Edition. (SCIDI/P). New York: Biometrics Research, New York State Psychiatric Institute

[B18] Gilboa E. Gotlib I. H. (1997). Cognitive biases and affect persistence in previously dysphoric and never-dysphoric individuals. Cogn. Emot. 11, 517–538 10.1080/026999397380032

[B19] Goldberg D. (1978). Manual of the General Health Questionnaire. Windsor: NFER-Nelson

[B20] Gotlib I. H. Cane D. B. (1987). Construct accessibility and clinical depression: a longitudinal investigation. J. Abnorm. Psychol. 96, 199–204 10.1037/0021-843X.96.3.199 3680757

[B21] Gupta R. Kar B. R. (2008). Interpretative bias: indicators of cognitive vulnerability to depression. German J. Psychiatry 11, 98–102

[B22] Hamilton M. A. (1960). Rating scale for depression. J. Neurol. Neurosurg. Psychiatr. 12, 56–62 10.1136/jnnp.23.1.56 14399272 PMC495331

[B23] Hammen C. L. Miklowitz D. J. Dyck D. G. (1986). Stability and severity parameters of depressive self-schema responding. J. Soc. Clin. Psychol. 4, 23–45 10.1521/jscp.1986.4.1.23

[B24] Hankin B. L. (2008). Stability of cognitive vulnerabilities to depression: a short-term perspective multiwave study. J. Abnorm. Psychol. 117, 324–333 10.1037/0021-843X.117.2.324 18489208 PMC2756216

[B25] Hedlund S. Rude S. S. (1995). Evidence of latent depressive schemas in formerly depressed individuals. J. Abnorm. Psychol. 104, 517–525 10.1037/0021-843X.104.3.517 7673575

[B26] Joormann J. Gotlib I. H. (2007). Selective attention to emotional faces following recovery from depression. J. Abnorm. Psychol. 116, 80–85 10.1037/0021-843X.116.3.484 17324018

[B27] Mansell W. Lam D. (2004). A preliminary study of autobiographical memory in remitted bipolar and unipolar depression and the role of imagery in the specificity of memory. Memory 12, 437–446 10.1080/09658210444000052 15487540

[B28] McCabe S. B. Gotlib I. H. (1993). Attentional processing in clinically depressed subjects: a longitudinal investigation. Cognit. Ther. Res. 17, 359–377 10.1007/BF01177660

[B29] Meites T. M. Deveney C. M. Steele K. T. Holmes A. J. Pizzagalli D. A. (2008). Implicit depression and hopelessness and remitted depressed individuals. Behav. Res. Ther. 46, 1078–1084 10.1016/j.brat.2008.05.008 18692169 PMC2630854

[B30] Nolen-Hoeksema S. (1991). Responses to depression and their effects on the duration of depressive episodes. J. Abnorm. Psychol. 100, 569–582 10.1037/0021-843X.100.4.569 1757671

[B31] Segal Z. V. Gemar M. (1997). Changes in cognitive organization for negative self-referent material following cognitive behavioral therapy for depression: a primes Stroop study. Cogn. Emot. 11, 501–516 10.1080/026999397379836a

[B32] Teasdale J. D. Dent J. (1987). Cognitive vulnerability to depression: an investigation of two hypotheses. Br. J. Clin. Psychol. 26, 113–126 10.1111/j.2044-8260.1987.tb00737.x 3580646

[B33] Watkins E. Teasdale J. D. Williams R. M. (2000). Decentering and distraction reduce overgeneral autobiographical memory in depression. Psychol. Med. 30, 911–920 10.1017/S0033291799002263 11037099

[B34] Williams J. M. Watts F. N. MacLeod C. Mathews A. (1997). Cognitive Psychology and Emotional Disorder. Chichester: Wiley

[B35] Williams J. M. G. Broadbent K. (1986). Autobiographical memory in attempted suicide patients. J. Abnorm. Psychol. 95, 144–149 10.1037/0021-843X.95.2.144 3711438

[B36] Williams J. M. G. Scott J. (1988). Autobiographical memory in depression. Psychol. Med. 18, 689–695 10.1017/S0033291700008515 3186869

